# *HOTTIP* polymorphism may affect gastric cancer susceptibility by altering HOTTIP expression

**DOI:** 10.1042/BSR20191687

**Published:** 2020-08-11

**Authors:** Ben-gang Wang, Yi-zhi Li, Han-xi Ding, Zhi Lv, Qian Xu, Yuan Yuan

**Affiliations:** 1 Tumor Etiology and Screening Department of Cancer Institute and General Surgery, The First Affiliated Hospital of China Medical University, and Key Laboratory of Cancer Etiology and Prevention (China Medical University), Liaoning Provincial Education Department, Shenyang 110001, China; 2 Hepatobiliary Surgery Department of General Surgery Institute, The First Affiliated Hospital of China Medical University, Shenyang 110001, China

**Keywords:** eQTLs, gastric cancer, HOTTIP, Single nucleotide polymorphism

## Abstract

***Background:*** Non-coding RNA polymorphisms can affect disease risk and prognosis by influencing gene expression. Here, we first investigated the association between single nucleotide polymorphisms (SNPs) of long non-coding RNA (lncRNA) *HOTTIP* and gastric cancer risk/prognosis. ***Methods:*** A total of five *HOTTIP* SNPs among 627 gastric cancer cases and 935 controls were tested by Kompetitive Allele Specific PCR (KASP) assay. The functional SNPs underwent eQTL analysis and the expression of HOTTIP was assessed by quantitative RT-PCR. ***Results:*** The rs2067087 and rs3807598 SNPs of *HOTTIP* increased susceptibility to gastric cancer (rs2067087: dominant model, *P*=0.008, odds ratio (OR) = 1.35; rs3807598: recessive model, *P*=0.037, OR = 1.29). Both *HOTTIP* rs2067087 and rs3807598 could affect the expression of mature lncRNA (*P*=0.003 and *P*=0.032, respectively). ***Conclusion:*** The rs2067087 and rs3807598 SNPs of *HOTTIP* are associated with gastric cancer risk, possibly by affecting the expression of mature HOTTIP.

## Introduction

Gastric cancer is the second most fatal type of tumor [1]. Patients with gastric cancer often respond poorly to treatment because of the heterogeneity of gastric cancer and limited treatment methods [2,3]. Therefore, the study of factors involved in early detection of gastric cancer and elucidation of the underlying mechanisms of gastric cancer pathogenesis are of significant interest.

Long non-coding RNAs (lncRNAs) are defined as transcripts containing more than 200 nucleotides, which are research hotspots particularly in oncology because of their wide biological regulatory functions [4]. LncRNAs play important roles in cancer pathogenesis by affecting diverse biological processes, including transcription, post-transcriptional regulation and epigenome [5–8]. So far, three key lncRNAs (HOTTIP, HOTAIR, and H19) have been reported to be candidate genes involved in carcinogenesis and potential therapeutic targets [9–14]. Among them, HOTTIP, transcribed from the 5′ tip of the HOXA cluster, is associated with various tumors including gastric cancer [15,16]. Recently, two studies have shown that HOTTIP is significantly overexpressed in gastric cancer cell lines and acts as a predictive factor for poor prognosis, suggesting that it may be a potential novel diagnostic and prognostic biomarker [17,18].

Genetic studies have shown that several single nucleotide polymorphisms (SNPs) are involved in increased susceptibility to gastric cancer [19–21]. Hu et al. showed that the functional *HOTTIP* rs1859168 A>C polymorphism might decrease the risk of pancreatic cancer [22]. However, the role of *HOTTIP* polymorphism in gastric cancer has not been investigated. With this in mind, we conducted the present study to identify functional SNPs in *HOTTIP* to determine any correlation of *HOTTIP* polymorphisms with gastric cancer susceptibility and prognosis, aiming to explore whether polymorphisms could affect the expression of mature HOTTIP.

## Materials and methods

### Patients and study design

This research project was approved by the Ethical Committee of the First Hospital of China Medical University and written informed consent was obtained. The study consisted of risk and prognosis studies, followed by eQTL analysis by quantitative RT-PCR for a step-by-step screening to find SNPs functional for gastric cancer etiology. This case–control study enrolled 1562 participants, including 627 gastric cancer patients and 935 matched controls. The patients received surgery for gastric cancer at the First Hospital of China Medical University between 2002 and 2013. The participants who had surgery were diagnosed with gastric cancer by pathological confirmation based on WHO classification. Then, 183 patients were diagnosed with intestinal-type gastric cancer and 312 with diffused-type gastric cancer according to Lauren classification. A total of 935 frequency-matched controls were recruited from a health-screening program from Zhuanghe, Liaoning Province, China, between 2002 and 2012 [23]. A questionnaire survey was conducted to collect information of smoking and drinking. We performed a follow-up visit for gastric cancer patients whose medical record was completed thereafter. The median survival time (MST) was 36 months and the last follow-up day was 1 July 2017.

### Selected SNP sites and genotyping

We selected polymorphic sites based on previous publication [24], which was shown in Supplementary Materials (Supplementary Figure S1 and Table S1). A total of five SNPs covering the *HOTTIP* gene were selected. Genomic DNA was extracted by a previously published method [25]. The genotyping assay was performed by Gene Company (Shanghai, China), using allele-specific PCR and Kompetitive Allele Specific PCR (KASP) reagents (LGC Genomics, Hoddesdon, U.K.) as previously described [24]. We repeated some samples for quality control, and the concordance rate reached more than 99% [24].

### Quantitative RT-PCR by eQTL analysis for HOTTIP expression and functional SNP identification

Approximately 50 mg total RNA was isolated from 39 gastric cancer specimens and 27 related cancer-free tissue using TRIzol reagent (Life Technologies, Carlsbad, CA, U.S.A.) as described in previous reports [24,26] shown in Supplementary Materials. The *HOTTIP* primer sequences were F: 5′-CGACTGGGTCCCTCCTCAC-3′ and R: 5′-GGCTCCTGCCGTCTTTTCT-3′. Analysis of eQTLs was performed by analyzing the effect of the polymorphisms on the lncRNA expression.

### Statistical analysis

Inter-group differences in sex variability and the Hardy–Weinberg equilibrium were compared by the Chi-squared (χ^2^) test, and the analysis of variance was performed for age variability. To evaluate the association between gene polymorphisms and gastric cancer risk, multivariate logistic regression adjusted for age and sex was used to calculate odds ratios (ORs) and their 95% confidence intervals (95% CIs). The haplotype of each gene was analyzed by SHEsis software [27]. The Student’s *t* test was used to test the differences in relative mRNA levels between the two groups. All statistical tests were two-sided and a *P*-value <0.05 was considered to be statistically significant.

## Results

### The association of HOTTIP SNPs with gastric cancer risk

The demographic information was presented in Supplementary Table S2. No significant difference was observed in either age or sex in gastric cancer cases and controls (*P*>0.05). Four SNPs (rs3807598, rs17501292, rs2067087, and rs17427960) of *HOTTIP* were accorded with the Hardy–Weinberg test (*P*>0.05), while rs78248039 was excluded because only the AA genotype was detected. Therefore, four SNPs were involved in the subsequent analysis.

By logistic regression analysis adjusted for age and gender, we found that two SNPs in *HOTTIP*, rs2067087 and rs3807598, were associated with gastric cancer risk. The dominant model of *HOTTIP* rs3807598 showed a 1.29-fold increased gastric cancer risk (*P*=0.037, 95% CI = 1.02–1.63) while the recessive model of *HOTTIP* rs2067087 showed a 1.35-fold increased gastric cancer risk (*P*=0.008, 95% CI = 1.08–1.68, Table 1). In addition, stratified analysis indicated that the patients with rs17501292 TG genotype were more likely to develop gastric cancer compared with the patients with TT genotype in the *H. pylori*-positive subgroup (*P*=0.022, OR = 4.12, 95% CI = 1.23–13.77, Supplementary Table S3). Female patients with rs2067087 CC *HOTTIP* genotype were more likely to have gastric cancer compared with the rs2067087 GG genotype (*P*=0.027, OR = 1.85, 95% CI = 1.07–3.19, Supplementary Table S3).

**Table 1:** The association of *HOTTIP* polymorphisms and gastric cancer risk

Gene	Chr. Pos.	SNP^1^	Loc.	Genotype	Controls (%)	Cases (%)	*P*^2^	OR (95% CI)	*P*_HWE_
HOTTIP	7p15.2	rs3807598	Exon 2	CC	257 (28.0)	143 (23.2)		1 (see footnote)	0.147
				CG	454 (49.4)	326 (52.9)	**0.043**	**1.29 (1.01–1.66)**	
				GG	208 (22.6)	147 (23.9)	0.108	1.27 (0.95**–**1.71)	
				CG+GG vs.CC			**0.037**	**1.29 (1.02–1.63)**	
				G vs.C			0.105	1.13 (0.98**–**1.30)	
		rs17501292	Exon 2	TT	853 (91.2)	567 (90.4)		1 (see footnote)	0.676
				TG	80 (8.6)	59 (9.4)	0.558	1.11 (0.78-1.58)	
				GG	2 (0.2)	1 (0.2)	0.846	0.79 (0.07**–**8.78)	
		rs2067087	Exon 2	GG	201 (21.6)	114 (18.4)		1 (see footnote)	0.390
				CG	467 (50.2)	291 (47.0)	0.498	1.10 (0.84**–**1.44)	
				CC	262 (28.2)	214 (34.6)	**0.014**	**1.44 (1.08–1.94)**	
				CC vs. GC+GG			**0.008**	**1.35 (1.08–1.68)**	
				C vs.G			**0.009**	**1.22 (1.05–1.40)**	
		rs17427960	Intron 2	CC	195 (21.2)	120 (19.8)		1 (see footnote)	0.122
				AC	446 (48.4)	278 (45.9)	0.926	1.01 (0.77**–**1.33)	
				AA	280 (30.4)	208 (34.3)	0.191	1.21 (0.91**–**1.62)	
		rs78248039	Exon 3	AA	877 (100)	576 (100)		1 (see footnote)	NA

Abbreviations: Chr. Pos., chromosomal position; Loc., localization; NA, not available; *P*_HWE_, *P*-value for Hardy–Weinberg equilibrium. ^1^, The sort order was according to the SNP location in its genes from 5′ starting to 3′ ends. ^2^, *P*-value was calculated by adjusted age and sex.

Regarding the Lauren classification, rs2067087 might be associated with the susceptibility to diffuse-type gastric cancer, as our analysis suggested that the CC genotype could increase the risk of diffuse-type gastric cancer compared with the GG genotype (*P*=0.046, OR = 1.48, 95% CI = 1.01–2.16, Table 2). However, in the further haplotype analysis, no haplotype of *HOTTIP* was found to be correlated with gastric cancer risk (Table 3).

**Table 2:** Association of *HOTTIP* polymorphisms with the risk of intestinal-type and diffuse-type gastric cancer

Gene	Variables	CON	Intestinal-type GC	Diffuse-type GC	Intestinal-type GC vs CON		Diffuse-type GC vs CON	
					*P*	OR (95% CI)^1^	*P*	OR (95% CI)^1^
HOTTIP	rs3807598							
	CC	257 (28.0)	44 (24.3)	71 (23.3)		1 (see footnote)		1 (see footnote)
	CG	454 (49.4)	95 (52.5)	156 (51.1)	0.301	1.23 (0.83–1.82)	0.189	1.24 (0.90–1.71)
	GG	208 (22.6)	42 (23.2)	78 (25.6)	0.512	1.17 (0.73–1.86)	0.097	1.37 (0.94–1.98)
	rs17501292							
	TT	853 (91.2)	160 (87.0)	284 (91.0)		1 (see footnote)		1 (see footnote)
	TG	80 (8.6)	23 (12.5)	28 (9.0)	0.111	1.50 (0.91–2.48)	0.784	1.07 (0.68–1.67)
	GG	2 (0.2)	1 (0.5)	0 (0)	0.492	2.41 (0.20–29.64)	NA	NA
	rs2067087							
	GG	201 (21.6)	35 (19.1)	53 (17.3)		1 (see footnote)		1 (see footnote)
	CG	467 (50.2)	76 (41.5)	153 (50)	0.708	0.92 (0.59–1.43)	0.219	1.25 (0.88–1.78)
	CC	262 (28.2)	72 (39.4)	100 (32.7)	0.056	1.55 (0.99–2.42)	**0.046**	**1.48 (1.01–2.16)**
	rs17427960							
	CC	195 (21.2)	34 (19.1)	59 (19.5)		1 (see footnote)		1 (see footnote)
	AC	446 (48.4)	78 (43.8)	146 (48.4)	0.925	0.98 (0.63–1.52)	0.624	1.09 (0.77–1.54)
	AA	280 (30.4)	66 (37.1)	97 (32.1)	0.211	1.34 (0.85–2.12)	0.417	1.17 (0.80–1.70)
	rs78248039							
	AA	877 (100)	171 (100)	285 (100)	NA	NA	NA	NA

Abbreviations: CON, control; GC, gastric cancer; NA, not available. The significance values were showed as bold font. ^1^, Using Logistic Regression adjusted by gender and age.

**Table 3:** The association of haplotype of *HOTTIP* and gastric cancer risk

Haplotype	Case (%)	Control (%)	*P*	OR (95% CI)
HOTTIP				
CTGC	475.65 (0.441)	763.41 (0.472)	0.086	0.87 (0.74–1.02)
GTCA	501.79 (0.465)	698.72 (0.432)	0.086	1.15 (0.98–1.36)

Using SHEsis software to analyze (http://analysis.bio-x.cn/).

### The association of HOTTIP SNPs with clinical parameters and prognosis of gastric cancer

We first analyzed the association of HOTTIP SNPs with clinical parameters of gastric cancer (Supplementary Table S4). In the univariate analysis of the clinical parameters and survival of gastric cancer patients, we found that the macroscopic type, TNM stage, depth of invasion, and lymphatic metastasis were associated with the survival time of gastric cancer patients (*P*<0.001, Supplementary Table S5). All the four clinical parameters were adjusted in the multivariate analysis of *HOTTIP* SNPs and gastric cancer prognosis. However, no statistical correlation between any of these four *HOTTIP* SNPs and gastric cancer prognosis were observed (Table 4).

**Table 4:** Univariate and multivariate Cox proportional hazard analyses for the association of *HOTTIP* polymorphisms and gastric cancer

Variables	SNP	genotype	All GC, *n* (%)	Deaths, *n*	MST^1^ (M)	Univariate		Multivariate^3^	
						*P*-value	Hazard ratio (95% CI)	*P*-value	Hazard ratio (95% CI)
HOTTIP	rs3807598		*n*=297	*n*=122					
		CC	63 (21.2)	26 (21.3)	55.3^2^		1 (see footnote)		1 (see footnote)
		CG	163 (54.9)	67 (54.9)	56.3^2^	0.802	1.06 (0.67–1.67)	0.747	1.08 (0.68–1.71)
		GG	71 (23.9)	29 (23.8)	68.0	0.990	1.00 (0.59–1.71)	0.703	1.11 (0.65–1.91)
	rs17501292		*n*=299	*n*=121					
		TT	273 (91.3)	110 (90.9)	57.5^2^		1 (see footnote)		1 (see footnote)
		TG	26 (8.7)	11 (9.1)	52.6^2^	0.869	1.05 (0.57–1.20)	0.429	0.78 (0.42–1.45)
		GG	NA	NA	NA	NA		NA	NA
	rs2067087		*n*=297	*n*=122					
		CC	97 (32.7)	44 (36.1)	68.0		1 (see footnote)		1 (see footnote)
		CG	151 (50.8)	58 (47.5)	58.7^2^	0.321	0.82 (0.55–1.21)	0.213	0.78 (0.53–1.15)
		GG	49 (16.5)	20 (16.4)	54.9^2^	0.556	0.85 (0.50–1.45)	0.610	0.87 (0.51–1.48)
	rs17427960		*n*=295	*n*=120					
		AA	98 (33.2)	42 (35)	68.0		1 (see footnote)		1 (see footnote)
		AC	144 (48.8)	57 (47.5)	58.0^2^	0.678	0.92 (0.62–1.37)	0.644	0.91 (0.61–1.36)
		CC	53 (18.0)	21 (17.5)	55.2^2^	0.745	0.92 (0.54–1.55)	0.687	0.90 (0.53–1.52)

Abbreviations: HR, hazard rate; NA: not available. ^1^, MST (months). ^2^, Mean survival time was provided when MST could not be calculated. ^3^, Multivariate survival analysis was carried out by adding the polymorphisms variable to the clinicopathological parameters with *P*<0.05.

### eQTL analysis

**Figure 1:**
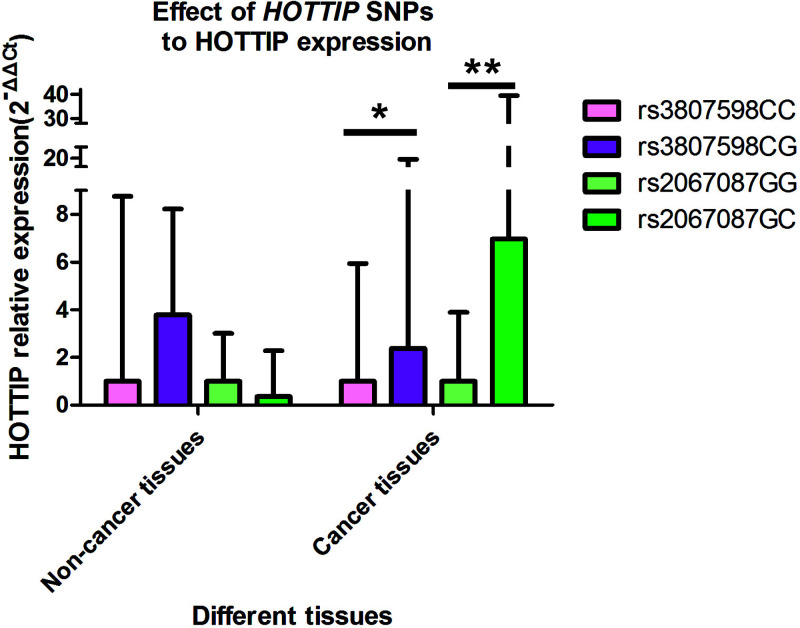
The effect of *HOTTIP*rs3807598 and rs2067087 polymorphisms on the HOTTIP corresponding mRNA expression *P=0.032, **P=0.003.

The impact of polymorphisms in *HOTTIP* on HOTTIP expression level was also analyzed. The HOTTIP expression level was significantly higher in the samples with heterozygous genotype than in wildtype samples for both *HOTTIP* rs3807598 and rs2067087 (*P*=0.032 and *P*=0.003, respectively, Table 5 and Figure 1).

**Table 5:** Differences of HOTTIP mRNA levels in different genotypes in gastric cancer and non-cancer tissues

Variable	Non-cancer tissue				Cancer tissue			
	*n*	Δ*C*_t_ (Mean ± SD)	Normalized 2^−ΔΔ*C*_t_^	*P*	*n*	Δ*C*_t_ (Mean ± SD)	Normalized 2^−ΔΔ*C*_t_^	*P*
HOTTIP	27	10.73 ± 2.28	1 (0.21, 4.86)	(see footnote)	39	11.59 ± 2.65	0.55 (0.09, 3.46)	0.331
Effect of HOTTIP rs3807598 genotypes on HOTTIP								
CC	3	11.21 ± 3.13	1 (0.11, 8.75)	(see footnote)	6	11.82 ± 2.20	1 (0.22, 5.94)	(see footnote)
GC	12	9.29 ± 1.12	3.78 (1.74, 8.22)	0.784	17	10.57 ± 3.02	2.38 (0.29, 19.29)	**0.032**
GG	7	10.25 ± 1.26	1.94 (3.24, 4.65)	0.370	7	13.85 ± 2.21	0.24 (0.05, 1.13)	0.125
GC+GG vs. CC	19	10.86 ± 2.59	1.27 (0.21, 7.67)	0.871	24	11.53 ± 3.16	1.22 (0.14, 10.93)	0.097
Effect of HOTTIP rs2067087 genotypes on HOTTIP								
GG	2	9.47 ± 1.59	1 (0.33, 3.01)		4	12.78 ± 1.96	1 (0.26, 3.89)	(see footnote)
GC	10	10.95 ± 2.67	0.36 (0.06, 2.28)	0.467	17	9.98 ± 2.50	6.96 (1.23, 39.40)	**0.003**
CC	10	10.70 ± 2.53	0.42 (0.07, 2.46)	0.788	11	10.51 ± 2.61	4.82 (0.79, 29.45)	0.907
CC+GC vs. GG	10	10.57 ± 2.56	0.47 (0.08, 2.75)	0.382	28	11.18 ± 2.81	3.63 (0.52, 25.46)	0.247

Abbreviation: NA, not available. *P*, the statistical analysis for the effect of genotype to phenotype was used two-independent sample *t* test, and for the combination of genotype to phenotype was used ANOVA analysis. Significance values are shown in bold.

## Discussion

Previous studies have demonstrated that overexpression of lncRNA HOTTIP in gastric cancer promotes tumor invasion and results in poor prognosis [17]. However, no investigation has focused on *HOTTIP* polymorphisms, which can affect HOTTIP expression in gastric cancer. The current study is the first report on the association between *HOTTIP* polymorphisms and gastric cancer. Here, we identified two functional SNPs associated with gastric cancer susceptibility that affect the expression of mature HOTTIP.

One of the two functional *HOTTIP* SNPs was rs2067087 with the minor allele of C. We found that rs2067087 statistically increased the risk of gastric cancer, and that female individuals carrying the rs2067087 CC genotype were more susceptible to gastric cancer compared with those with GG genotype. This suggested that determining the rs2067087 genotype might be a potentially meaningful test in gastric cancer screening, especially for females. In a previous study, we found that the variant genotype of rs2067087 could increase hepatocellular cancer (HCC) risk [24]. The rs2067087 polymorphism is located in an exon of the *HOTTIP* gene and can combine with some functional proteins. Through a CHIP-Seq experiment, this SNP was found to bind to SUZ12 protein [28], which promoted gastric cancer cell invasion [29]. Although the detailed mechanisms require further study, the rs2067087 polymorphism may be a functional SNP participating in gastric carcinogenesis and a candidate biomarker for the prediction of gastric cancer.

Another *HOTTIP* SNP detected to play a role in gastric carcinogenesis was rs3807598. It was suggested that the heterozygous genotype had a significantly increased risk of gastric cancer, and a significant association under the dominant model (GG+CG/CC) between rs3807598 and the risk of gastric cancer was observed. Although no statistical difference in the stratified analysis of rs3807598 was found considering gender, age, cigarette or alcohol consumption, and *Helicobacter pylori* infection status, this allele might serve as a risk marker of gastric cancer. Further study focused on the detailed molecular mechanism of rs3807598 in gastric cancer is required.

We also carried out genotype–phenotype analysis and observed that both the heterozygous genotypes of rs3807598 and rs2067087 associated with gastric cancer susceptibility contributing to higher HOTTIP expression than wildtype SNPs. A previous study showed that the rs1859168 A>C polymorphism regulated HOTTIP expression and reduced the risk of pancreatic cancer in a Chinese population [22]. In our study, we identified two SNPs (rs3807598 and rs2067087) in *HOTTIP* involved in gastric cancer susceptibility and the formation of mature HOTTIP. In recent years, Harrow et al. showed that the rs2067087 SNP could combine with SUZ12 protein [28], while Westra et al. found that another SNP, rs3807598, had an effect as trans-eQTL, acting as a putative driver in whole blood with a significant *P*-value (*P*=0.00019) [30]. Here, we found that the risk-associated heterozygous genotypes of these two SNPs showed higher HOTTIP expression. Because HOTTIP functions as an oncogenic lncRNA, we speculate that the risk-associated rs3807598 and rs2067087 SNPs could participate in gastric carcinogenesis by up-regulating the expression of mature HOTTIP. Further molecular experiments should be performed to verify our results.

In view of the impact of *HOTTIP* polymorphisms on the overall survival of patients with gastric cancer, we found that, in this case–control study, none of these four SNPs affected the prognosis of gastric cancer. We previously reported that HOTTIP overexpression was associated with poor gastric cancer prognosis [17], and Ye et al. identified HOTTIP expression levels as an independent factor for poor prognosis in gastric cancer patients [17]. Although both the heterozygous genotypes of rs3807598 and rs2067087 up-regulated HOTTIP expression, either rs3807598 or rs2067087 heterozygous genotype was not significantly correlated with poor survival. Further studies are needed to investigate the association between *HOTTIP* SNPs and the prognosis of gastric cancer.

It should be pointed that the study had some limitations. First, the sample size was limited, causing limited probability of the stratified and interaction analysis for variant genotypes. Second, the expression data of lncRNA-HOTTIP gene in the present study was only based on RNA level. Experiments *in vitro* are needed in future research. Third, it is a relatively peripheral association study and lacking functional evidence that links to the studied SNPs and HOTTIP. Therefore, further confirmation would be warranted.

## Conclusion

The present study first identified two functional SNPs (rs3807598 and rs2067087) in *HOTTIP* with the potential to predict gastric cancer risk. These SNPs variants were associated with corresponding HOTTIP expression, providing clues for further studies focused on HOTTIP SNPs and gastric cancer pathogenesis. For the future perspective, lncRNA SNPs could have potential to be biomarkers for gastric cancer risk and help to elucidate the etiology of gastric cancer.

## Supplementary Material

Supplementary Figure S1 and Tables S1-S5

## Abbreviations

CI: confidence interval
eQTL: expression quantitative trait Loci
lncRNA: long non-coding RNA
MST: median survival time
OR: odds ratio
SNP: single nucleotide polymorphism
TNM: Tumor Node Metastasis
