# Supplementary material for: HOTTIP polymorphism may affect gastric cancer susceptibility by altering HOTTIP expression
Source: Biosci Rep. 2020 Aug 11;40(8):BSR20191687. doi: 10.1042/BSR20191687 (PMC7419746; doi:10.1042/BSR20191687)
Supplement: Supplementary Figure S1 and Tables S1-S5 [file BSR-2019-1687_supp.pdf]

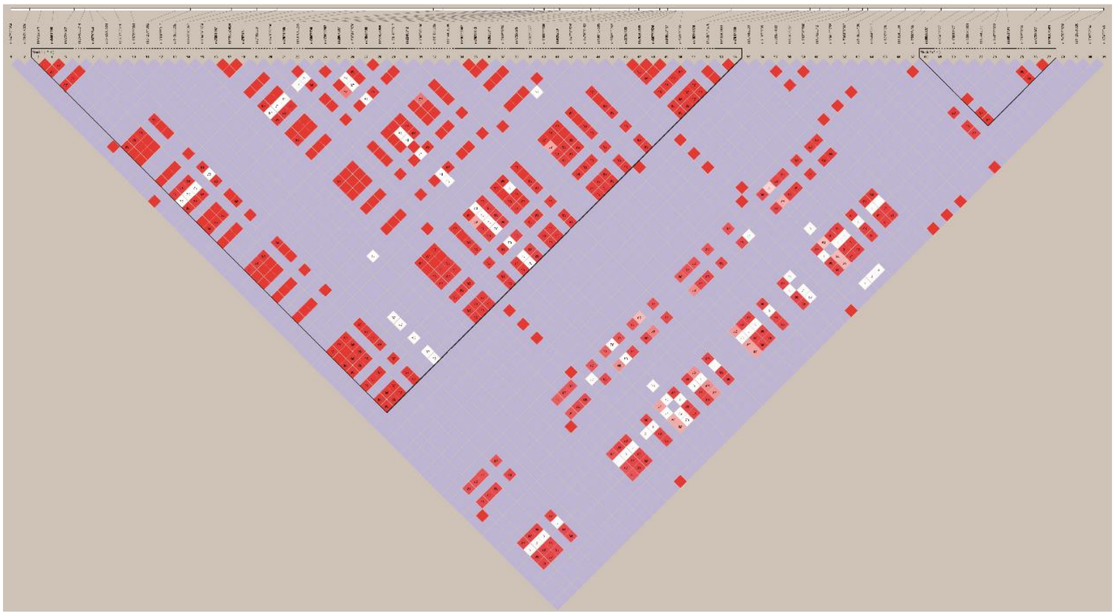

Supplementary Figure 1. The LD figure of tagSNPs of *HOTTIP* gene

Supplementary Table S1. The selected SNP and captured others

Test	Alleles Captured
rs2067087	rs61056117, rs4722672, rs2023843, rs115525024, rs1859168, rs2071265, rs7812039, rs6461995, rs2391397, rs2023844, rs2240042, rs929250, rs1859168, rs3735533, rs4722671, rs10228276, rs60772526, rs4722675, rs9770544, rs757181, rs13243033
rs3807598	rs3807598, rs10233387
rs17427960	rs17427960
rs78248039	rs78248039
rs17501292	rs17501292

Supplementary Table S2. The baseline of the subjects

Variables	CON (%)		GC (%)
	**n=953**		**n=631**
Gender		*P*=0.101	
Male	641(67.3)		449(71.2)
Female	312(32.7)		182(28.8)
Age		*P*=0.568	
Mean±SD	58.9±10.3		59.2±11.4
Median	58		59
Range	26-90		26-87
H.pylori		*P*＜0.001	
Positive	106(16.6)		320(51.1)
Negative	531(83.4)		306(48.9)
Smoking		*P*=0.120	
Ever Smoker	231(34.0)		149(42.0)
Never Smoker	448(66.0)		206(58.0)
Drinking		*P*＜0.001	
Drinker	120(17.7)		115(35.5)
Nondrinker	557(82.3)		209(64.5)

Supplementary Table S3. Association of lnRNA polymorphisms with the risk of gastric cancer stratified by host characteristics

Variables	Genotype	GC vs CON	*P*	OR (95%CI)
HOTTIP rs3087598				
Gender				
Male	CC	106/175		1(Ref)
	CG	224/307	0.245	1.19(0.89-1.61)
	GG	108/141	0.199	1.26(0.89-1.78)
Female	CC	37/82		1(Ref)
	CG	102/147	0.078	1.52(0.95-2.42)
	GG	39/67	0.422	1.26(0.72-2.20)
Age				
≦60	CC	79/149		1(Ref)
	CG	183/265	0.141	1.28(0.92-1.79)
	GG	77/118	0.314	1.23(0.83-1.82)
>60	CC	64/108		1(Ref)
	CG	143/189	0.235	1.26(0.86-1.85)
	GG	70/90	0.301	1.27(0.81-1.98)
Smoking				
Ever smoker	CC	41/67		1(Ref)
	CG	68/106	0.891	1.04(0.63-1.71)
	GG	37/51	0.565	1.19(0.66-2.13)
Never smoker	CC	42/117		1(Ref)
	CG	111/219	0.111	1.41(0.92-2.16)
	GG	50/93	0.118	1.50(0.90-2.49)
Alcohol drinking				
Drinker	CC	29/30		1(Ref)
	CG	53/55	0.982	1.01(0.53-1.92)
	GG	28/31	0.857	0.94(0.45-1.92)
Nondrinker	CC	45/153		1(Ref)
	CG	111/268	0.098	1.40(0.94-2.09)
	GG	51/114	0.078	1.53(0.95-2.45)
H.pylori[a]				
negative	CC	67/144		1(Ref)
	CG	163/257	0.100	1.34(0.95-1.91)
	GG	90/112	0.156	1.35(0.89-2.05)
positive	CC	74/21		1(Ref)
	CG	161/55	0.479	0.81(0.46-1.45)
	GG	76/26	0.519	0.80(0.41-1.56)
HOTTIP rs17501292				
Gender				
Male	TT	407/574		1(Ref)
	TG	38/56	0.812	0.95(0.62-1.46)
	GG	1/0	NA	NA
Female	TT	160/279		1(Ref)
	TG	21/24	0.168	1.55(0.83-2.88)
	GG	0/2	NA	NA
Age				
≦60	TT	313/497		1(Ref)
	TG	33/41	0.315	1.28(0.79-2.07)

	GG	0/0	NA	NA
>60	TT	254/356		1(Ref)
	TG	26/39	0.855	0.95(0.56-1.61)
	GG	1/2	0.924	0.89(0.08-10.19)
Smoking				
Ever smoker	TT	128/205		1(Ref)
	TG	20/21	0.298	1.42(0.73-2.75)
	GG	0/0	NA	NA
Never smoker	TT	194/398		1(Ref)
	TG	11/39	0.137	0.59(0.29-1.18)
	GG	0/2	NA	NA
Alcohol drinking				
Drinker	TT	103/108		1(Ref)
	TG	11/9	0.603	1.28(0.51-3.23)
	GG	0/0	NA	NA
Nondrinker	TT	190/493		1(Ref)
	TG	18/51	0.725	0.90(0.51-1.59)
	GG	0/2	NA	NA
H.pylori[a]				
negative	TT	276/472		1(Ref)
	TG	26/50	0.630	0.89(0.54-1.46)
	GG	1/1	0.730	1.63(0.10-26.46)
positive	TT	186/102		1(Ref)
	TG	33/3	**0.022**	**4.12(1.23-13.77)**
	GG	0/0	NA	NA
HOTTIP rs2067087				
Gender				
Male	GG	85/134		1(Ref)
	CG	209/315	0.847	1.03(0.75-1.43)
	CC	148/179	0.150	1.29(0.91-1.83)
Female	GG	29/67		1(Ref)
	CG	82/152	0.442	1.22(0.73-2.05)
	CC	66/83	**0.027**	**1.85(1.07-3.19)**
Age				
≦60	GG	49/86		1(Ref)
	CG	131/186	0.487	1.16(0.76-1.78)
	CC	99/119	0.108	1.44(0.92-2.24)
>60	GG	65/115		1(Ref)
	CG	160/281	0.998	1.00(0.69-1.43)
	CC	115/143	0.081	1.42(0.96-2.10)
Smoking				
Ever smoker	GG	31/50		1(Ref)
	CG	63/110	0.732	0.91(0.52-1.58)
	CC	54/67	0.458	1.25(0.70-2.23)
Never smoker	GG	36/93		1(Ref)
	CG	99/225	0.547	1.15(0.73-1.82)
	CC	68/118	0.107	1.51(0.92-2.48)
Alcohol drinking				
Drinker	GG	24/22		1(Ref)
	CG	50/59	0.408	0.74(0.37-1.50)
	CC	39/36	0.959	0.98(0.47-1.05)
Nondrinker	GG	36/120		1(Ref)
	CG	101/274	0.336	1.24(0.80-1.92)

	CC	70/150	0.067	1.55(0.97-2.49)
H.pylori[a]				
negative	GG	53/110		
	CG	141/262	0.587	1.11(0.76-1.64)
	CC	106/147	0.060	1.49(0.98-2.25)
positive	GG	59/20		
	CG	148/57	0.619	0.86(0.47-1.56)
	CC	107/27	0.423	1.31(0.68-2.55)
HOTTIP rs17427960				
Gender				
Male	CC	88/133		1(Ref)
	AC	197/300	0.889	0.98(0.71-1.35)
	AA	146/188	0.389	1.16(0.82-1.65)
Female	CC	32/62		1(Ref)
	AC	81/146	0.785	1.07(0.65-1.78)
	AA	62/92	0.282	1.35(0.78-2.31)
Age				
≦60	CC	94/123		1(Ref)
	AC	127/185	0.995	1.00(0.67-1.52)
	AA	68/116	0.518	1.16(0.74-1.81)
>60	CC	68/116		1(Ref)
	AC	151/261	0.891	0.98(0.68-1.40)
	AA	114/157	0.288	1.23(0.84-1.81)
Smoking				
Ever smoker	CC	37/50		1(Ref)
	AC	53/104	0.148	0.67(0.38-1.17)
	AA	53/70	0.921	1.03(0.59-1.81)
Never smoker	CC	34/85		1(Ref)
	AC	98/214	0.603	1.13(0.71-1.81)
	AA	67/131	0.366	1.26(0.76-2.09)
Alcohol drinking				
Drinker	CC	24/19		1(Ref)
	AC	46/55	0.167	0.59(0.28-1.25)
	AA	41/38	0.628	0.83(0.39-1.76)
Nondrinker	CC	39/115		1(Ref)
	AC	98/261	0.638	1.11(0.72-1.71)
	AA	66/164	0.471	1.19(0.75-1.89)
H.pylori[a]				
negative	CC	55/106		1(Ref)
	AC	140/255	0.816	1.05(0.71-1.54)
	AA	100/152	0.262	1.27(0.84-1.92)
positive	CC	63/20		1(Ref)
	AC	136/51	0.513	0.82(0.45-1.49)
	AA	107/31	0.861	1.06(0.55-2.03)

Note: [a] using Logistic Regression adjusted by the other two factors of gender, age and *H.pylori* infection status. [b] using Logistic Regression adjusted by gender, age and *H.pylori* infection status. CON: controls; AG: atrophic gastritis; GC: gastric cancer. NA, not available.

Supplementary Table S4. The associated of HOTTIP SNPs and clinical parameters of gastric cancer

Parameters	Genotypes			Heterozygote vs. wild-type	Mutation vs. wild-type	Dominant model	Recessive model
	Wild-type	Heterozygote	Mutation-type	*P-value*	*P-value*	*P-value*	*P-value*
rs3807598							
Age				0.424	0.243	0.312	0.390
≤60	40(63.5)	94(57.7)	38(53.5)				
＞60	23(36.5)	69(42.3)	33(46.5)				
Sex				0.211	0.312	0.204	0.788
Male	50(79.4)	116(71.2)	51(71.8)				
Female	13(20.6)	47(28.8)	20(28.2)				
Macroscopic type				0.077	0.286	0.108	0.294
Early stage	4(6.3)	16(9.8)	10(14.1)				
Borrmann Ⅰ-Ⅱ	10(15.9)	46(28.2)	13(18.3)				
BorrmannⅢ-Ⅳ	49(77.8)	101(62)	48(67.6)				
Lauren grade				**0.012**	0.116	**0.015**	0.931
Intestinal	31(49.2)	51(31.3)	25(35.7)				
Diffuse	32(50.8)	112(68.7)	45(64.3)				
TNM stage				0.754	0.992	0.816	0.815
Ⅰ-Ⅱ	32(50.8)	79(48.5)	36(50.7)				
Ⅲ-Ⅳ	31(49.2)	84(51.5)	35(49.3)				
Growth pattern				0.366	0.822	0.589	0.328
Massive/Nested	25(46.3)	48(39.0)	30(48.4)				
Diffused	29(53.7)	75(61.0)	32(51.6)				
Depth of invasion				0.407	0.414	0.366	0.700

T1+T2	24(38.1)	72(44.2)	32(45.1)				
T3+T4	39(61.9)	91(55.8)	39(54.9)				
Lymphatic metastasis				0.912	0.977	0.944	0.901
Positive	38(60.3)	97(59.5)	43(60.6)				
Negative	25(39.7)	66(40.5)	28(39.4)				
H. pylori-IgG				0.480	0.726	0.699	0.321
Positive	35(55.6)	81(50.3)	41(58.6)				
Negative	28(44.4)	80(49.7)	29(41.4)				
rs17501292							
Age				0.664			
≤60	159(58.2)	14(53.8)	NA				
＞60	114(41.8)	12(46.2)	NA				
Sex				**0.001**			
Male	207(75.8)	12(46.2)	NA				
Female	66(24.2)	14(53.8)	NA				
Macroscopic type				0.505			
Early stage	29(10.6)	1(3.8)	NA				
Borrmann Ⅰ-Ⅱ	65(23.8)	5(19.2)	NA				
BorrmannⅢ-Ⅳ	179(65.6)	20(76.9)	NA				
Lauren grade				0.289			
Intestinal	97(35.7)	12(46.2)	NA				
Diffuse	175(64.3)	14(53.8)	NA				
TNM stage				0.695			
Ⅰ-Ⅱ	137(50.2)	12(46.2)	NA				
Ⅲ-Ⅳ	136(49.8)	14(53.8)	NA				
Growth pattern				0.295			
Massive/Nested	94(42.9)	12(54.5)	NA				

Diffused	125(57.1)	10(45.5)	NA				
Depth of invasion				0.340			
T1+T2	121(44.3)	9(34.6)	NA				
T3+T4	152(55.7)	17(65.4)	NA				
Lymphatic metastasis				0.548			
Positive	162(59.3)	17(65.4)	NA				
Negative	111(40.7)	9(34.6)	NA				
*H. pylor*i-IgG				0.691			
Positive	146(54.1)	13(50)	NA				
Negative	124(45.9)	13(50)	NA				
rs2067087							
Age				0.645	0.319	0.476	0.378
≤60	53(54.6)	87(57.6)	31(63.3)				
>60	44(45.4)	64(42.4)	18(36.7)				
Sex				0.416	**0.010**	0.120	**0.015**
Male	66(68)	110(72.8)	43(87.8)				
Female	31(32)	41(27.2)	6(12.2)				
Macroscopic type				0.296	0.744	0.340	0.800
Early stage	12(12.4)	12(7.9)	4(8.2)				
Borrmann Ⅰ-Ⅱ	19(19.6)	40(26.5)	10(20.4)				
BorrmannⅢ-Ⅳ	66(68)	99(65.6)	35(71.4)				
Lauren grade				0.386	0.055	0.994	**0.008**
Intestinal	35(36.5)	47(31.1)	26(53.1)				
Diffuse	61(63.5)	104(68.9)	23(46.9)				
TNM stage				0.545	0.597	0.507	0.775
Ⅰ-Ⅱ	45(46.4)	76(50.3)	25(51)				
Ⅲ-Ⅳ	52(53.6)	75(49.7)	24(49)				

Growth pattern				0.127	0.749	0.283	0.263
Massive/Nested	39(48.1)	44(37.3)	21(51.2)				
Diffused	42(51.9)	74(62.7)	20(48.8)				
Depth of invasion				0.868	0.601	0.961	0.504
T1+T2	42(43.3)	67(44.4)	19(38.8)				
T3+T4	55(56.7)	84(55.6)	30(61.2)				
Lymphatic metastasis				0.433	0.577	0.416	0.824
Positive	62(63.9)	89(58.9)	29(59.2)				
Negative	35(41.1)	62(41.1)	20(40.8)				
*H. pylor*i-IgG				0.987	0.821	0.947	0.794
Positive	51(53.1)	79(53)	27(55.1)				
Negative	45(46.9)	70(47)	22(44.9)				
rs17427960							
Age				0.611	0.473	0.895	0.289
≤60	41(41.8)	79(54.9)	34(64.2)				
＞60	57(58.2)	65(45.1)	19(35.8)				
Sex				0.375	0.092	0.184	0.151
Male	67(68.4)	106(73.6)	43(81.1)				
Female	31(31.6)	38(26.4)	10(18.9)				
Macroscopic type				0.345	0.483	0.347	0.477
Early stage	13(13.3)	12(8.3)	4(7.5)				
Borrmann Ⅰ-Ⅱ	21(21.4)	39(27.1)	10(18.9)				
BorrmannⅢ-Ⅳ	64(65.3)	93(64.6)	39(73.6)				
Lauren grade				0.345	0.156	0.857	0.034
Intestinal	36(37.1)	45(31.3)	26(49.1)				
Diffuse	61(62.9)	99(68.7)	27(50.9)				
TNM stage				0.497	0.638	0.484	0.858

Ⅰ - Ⅱ	46(46.9)	74(51.4)	27(50.9)				
Ⅲ-Ⅳ	52(53.1)	70(48.6)	26(49.1)				
Growth pattern				0.064	0.905	0.147	0.414
Massive/Nested	40(50)	41(36.6)	22(48.9)				
Diffused	40(50)	71(63.4)	23(51.1)				
Depth of invasion				0.573	0.402	0.896	0.221
T1+T2	42(44.1)	67(46.5)	19(35.8)				
T3+T4	56(53.9)	77(53.5)	34(64.2)				
Lymphatic metastasis				0.441	0.565	0.419	0.804
Positive	62(63.3)	84(58.3)	31(58.5)				
Negative	36(36.7)	60(41.7)	22(41.5)				
*H. pylor*i-IgG				0.738	0.565	0.965	0.414
Positive	52(53.6)	73(51.4)	31(58.5)				
Negative	45(46.4)	69(48.6)	22(41.5)				

NA, not available.

Supplementary Table S5. Gastric cancer patient clinical features and univariate analysis of overall survival

Factors	All GC N (%)	Death, n	MST[a] (M)	*P-value*
Macroscopic type	n=301	n=122		
Borrmann Ⅰ	31(10.3)	4(3.3)	72.0[b]	**0.001**
Borrmann Ⅱ	70(23.3)	22(18.0)	65.1[b]	
BorrmannⅢ+Ⅳ	200(66.4)	96(78.7)	53.0	
Ⅰ+Ⅱ vs. Ⅲ+Ⅳ				NA
Lauren grade	n=300	n=122		
Intestinal	109(36.3)	42(34.4)	56.7[b]	0.395
Diffuse	191(63.7)	80(65.6)	79.0	
TNM stage	n=301	n=122		
Ⅰ	64(21.2)	3(2.5)	79.2[b]	**2.6×10^{-13}**
Ⅱ	86(28.6)	22(18.0)	65.4[b]	
Ⅲ	124(41.2)	76(62.3)	29.0	
Ⅳ	27(9.0)	21(17.2)	15.0	
Ⅰ+Ⅱ vs. Ⅲ+Ⅳ				0.001
Depth of invasion	n=301	n=122		
T1+T2	131(43.5)	22(18.0)	75.4[b]	**1.9×10^{-12}**
T3+T4	170(56.5)	100(82.0)	30.0	
Lymphatic metastasis	n=301	n=122		
Negative	121(40.2)	22(18.0)	70.1[b]	**4.5×10^{-9}**
Positive	180(59.8)	100(82.0)	36.0	
Smoking	n=248	n=103		
Never Smoker	149(60.1)	63(61.2)	53.2b	0.815
Ever Smoker	99(39.9)	40(38.8)	79.0	
Drinking	n=248	n=103		
Nondrinker	167(67.3)	69(67.0)	54.0b	0.993
Drinker	81(32.7)	34(33.0)	79.0	
Family history	n=244	n=102		
No	211(86.5)	90(88.2)	79.0	0.448
Yes	33(13.5)	12(11.8)	58.2[b]	

[a], MST, median survival time (months). [b], mean survival time was provided when MST could not be calculated.

Supplementary Materials and Methods

We used the HapMap download function of Haploview 4.2 software. TagSNPs for the *HOTTIP* gene were selected separately by Tagger within Haploview using the following criteria: pairwise tagging of the HapMap population with r^2 of at least 0.8; a minor allele frequency (MAF) of at least 5%; and Chinese Han Beijing (CHB) ethnicity. We expanded 10kbp from *HOTTIP* gene upstream. The linkage disequilibrium (LD) of *HOTTIP* gene was shown in Supplementary Figure 1. Then five SNPs covered *HOTTIP* genewere selected, other SNPs captured by the selected ones were shown in Supplementary Table S4.

1.5ug of isolated total RNA was converted into cDNA using Quantscript RT Kit (Tiangen Biotech, Beijing, China). The mRNA levels specific for*HOTTIP* gene and an internal-control gene *GAPDH* were examined using SYBR Premix Ex Taq II (TaKaRa Biotech, Dalian, China) in an Eppendorf Mastercycler Gradient System (Eppendorf AG, Hamburg, Germany) according to the manufacture's protocol. Melting curve analysis was performed to exclude the presence of non-specific products and primer-dimers. Each reaction was performed in duplicates and no-template controls were included in each experiment.

The relative quantification of gene mRNA expression was calculated using the $2^{-\Delta\Delta Ct}$ method [1]. The expression levels of*HOTTIP*were normalized to those of *GAPDH* in each sample using the equation: ΔCt(delta Ct)=Ct_{target}-Ct_{GAPDH}. Relative expression levels were derived from ΔCt-values as $2^{-\Delta Ct}$. For patients stratified by polymorphic genotypes, the relative expression levels of the common homogenous genotype carriers were set to a unity, and the relative expression levels of patients with heterogeneous and rare homogenous genotypes were expressed relative to those of the common homogenous genotype carriers, thus deriving normalized $2^{-\Delta\Delta Ct}$ values.

Reference

1 Livak KJ, Schmittgen TD. Analysis of relative gene expression data using real-time quantitative PCR and the 2(-Delta Delta C(T)) Method. *Methods (San Diego, Calif* 2001; **25**(4): 402-408 [PMID: 11846609]
